# Prevalence of sexually transmitted infections in women of the Czech Republic Armed Forces: a cross-sectional pilot study

**DOI:** 10.1136/military-2023-002611

**Published:** 2024-05-06

**Authors:** Lubos Karasek, J Smetana, P Svobodova, J Smahelova, R Tachezy, I Kiss, D Nejedla

**Affiliations:** 1 Department of Epidemiology, Military Faculty of Medicine, University of Defence, Hradec Kralove, Czech Republic; 2 Department of Gynecology 3rd Faculty of Medicine of Charles University and Military University Hospital Prague, Military University Hospital Prague, Prague, Czech Republic; 3 Department of Genetics and Microbiology, Faculty of Science, Charles University, BIOCEV, Vestec, Czech Republic; 4 Department of Microbiology, Military University Hospital Prague, Prague, Czech Republic

**Keywords:** EPIDEMIOLOGY, Epidemiology, Infection control, VIROLOGY, Infectious diseases & infestations

## Abstract

**Introduction:**

Sexually transmitted infections (STIs) are an everlasting health issue globally. The military environment is recognised as a high-risk setting. Human papillomavirus (HPV), *Chlamydia trachomatis* and *Neisseria gonorrhoeae* are the most frequent STIs worldwide. This prospective cross-sectional pilot study focuses on the prevalence of selected STIs in the female population of the Czech Republic’s Armed Forces.

**Methods:**

*C. trachomatis*, *N. gonorrhoeae* and HPV detection and genotyping were performed between August 2020 and December 2022 in 141 women. Participants were divided into three groups according to their military status—recruits (n=72), active soldiers (n=25) and control civilian group (n=44). Cervical smear tests were performed, and data on STI risk factors were obtained through a questionnaire.

**Results:**

A significant difference in the HPV prevalence between recruits (64.5 %) and both active soldiers (46.4 %) and civilians (47.3 %) was found when adjusted for age (p=0.007 and p=0.01, respectively). Lower age of coitarche (median 16; p=0.005) and smaller agglomeration origin (p=0.013) were reported for military recruits. No difference was proven in other researched risk factors. Associations between HPV detection and the higher number of sexual partners (p=0.013), early coitarche (p=0.016) and single marital status (p=0.002) across the groups were observed. Not a single case of *N. gonorrhoeae* was detected in any of the 141 participants. The prevalence of *C. trachomatis* did not differ significantly between the three evaluated groups—recruits, control civilian group, and active soldiers (5.6%, 2.3%, 0%, respectively; p=0.567).

**Conclusions:**

This pilot study showed a significantly higher HPV prevalence in female military recruits compared with both active military and civilian women. Recruits reported earlier coitarche which is a strong STI risk factor. Further study is needed to expand on the findings of this pilot study and generate data to support adjustment of STI preventive measures within the Czech Republic Armed Forces.

**WHAT IS ALREADY KNOWN ON THIS TOPIC**

- Military service is perceived as a risky environment in terms of sexually transmitted infections (STIs), and a higher prevalence of STIs in the military was shown in several studies.

**WHAT THIS STUDY ADDS**

- This study researched human papillomavirus (HPV), *Chlamydia trachomatis* and *Neisseria gonorrhoeae* prevalence in women of the Czech Republic Armed Forces and did find a significantly higher HPV prevalence in military recruits when compared with women of Armed Forces and the general population.
- The group of recruits seems to be at higher risk of acquiring STIs—suggesting that further research would be appreciated to confirm the evidence.

**HOW THIS STUDY MIGHT AFFECT RESEARCH, PRACTICE OR POLICY**

- Preventive measures against STIs across the military forces should be considered.

## Introduction

 Sexually transmitted infections (STIs) are a frequent and everlasting challenge for both individuals and society at large, possibly causing serious health issues. Human papillomavirus (HPV) and *Chlamydia trachomatis* are perceived as the most frequent ones. HPVs can be divided into two large subgroups: low-risk HPV (LR HPV) causing benign skin and mucosal warts and high-risk HPV (HR HPV) being the main cause of anogenital and epithelial dysplasia and cancer, most frequently cervical and in the last decades also oropharyngeal cancer.^1^,^3^ *C. trachomatis* is a common cause of urogenital infections and subsequently also deteriorated fertility.^4 5^ *Neisseria gonorrhoeae* is not as frequent of a pathogen as those mentioned earlier, but the clinical consequences of its infection could be serious, and the frequency in the population is rising.^6^

Military service is perceived as a risky environment in matters of STIs. Historically, this poor reputation has been attributed to specific life conditions, minimal knowledge of the STI pathogenesis and the absence of preventive measures.^7^ With advances in medicine such as diagnostic testing, therapeutic options and health education, and major changes in the military forces conception, the unfavourable epidemiology of the STIs in Armed Forces has changed.^8^ The risk of acquiring STI in the army is lower than in the past. However, data show that the prevalence of STIs is still higher compared with the general population.^9^,^11^ The prevalence of the STIs in the army is not only dependent on the military environment, but it is also influenced by the STI status of members joining the military service. Agan *et al* showed that HPV seroprevalence among male military recruits was higher than that reported among civilian men.^12^ Most reliable data on this matter come from the US military; however, data available from Central Europe are scarce.^13 14^ Furthermore, the available data are largely limited to male populations despite women being at greater risk of STIs.^15^

No data on STIs are available for the Army of the Czech Republic. To date, there is no mandatory screening for STIs, either at the time of entry or throughout the military service period. Considering the higher risk of STIs posed to women, we aimed to evaluate the epidemiology of common STIs in female recruits and soldiers of the Czech Republic army and compare prevalence with a sample from the civilian population.

## Methods

The prospective cross-sectional pilot study was performed in theMilitary University hospital Prague between August 2020 and December 2022. Women ranging in age between 18 years and 40 years (N=141), formed the part of the study. They represented three different population groups, following the groups compared in the study. The first group (G1) comprised recruits, women who were in the process of entering the army and visiting the gynaecology outpatient department as part of their general medical entry evaluation/screening. The second group (G2) comprised female soldiers undergoing an annual gynaecological examination. The third group (G3), stated as the control group, constituted of civilian women without connection to the army, also undergoing an annual routine examination in the Military University Hospital Prague. Participation in the study was voluntary. The participants were informed about the study protocol including vaginal examination, and a written consent was obtained. Subjects’ data were numerically pseudoanonymised. Subsequently, a follow-up of women with positive STIs’ test was provided. An appropriate antibiotic regimen was prescribed in cases of *C. trachomatis* positivity, and an additional Pap smear and colposcopy were recommended to HR HPV-positive participants. Exclusion criteria were stated as recently (1 year or less) known and/or treated STI infection and dysplastic or neoplastic gynaecological conditions of the vulva, vagina and uterine cervix or body. Women with a history of hysterectomy or trachelectomy were also excluded from the study. Patients with cured vulvar, vaginal, cervical or uterine disease and/or a medical history of conisation or other forms of therapy for cervical disease more than a year ago were included in the study.

### Specimen collection and processing

Samples were taken from women during a routine gynaecological examination. Two smears were taken from the uterine cervix, covering both endocervix and exocervix, using three brush rotations. First, a mini tip brush in a kit with liquid amies preservation medium eSwab (Copan, Brescia, Italy) was used for *C. trachomatis* and *N. gonorrhoeae*. Then the smear for HPV detection was performed by a FLOQSwabs brush (Copan) and inserted into the transport tube with 2 mL PreservCyt solution (Roche, Rotkreuz, Switzerland). Samples were taken and handled by two similarly experienced clinicians using a standardised sampling technique. Both specimen tubes were then stored at a temperature of −20°C until the laboratory processing.

At the enrolment to the study, women were asked to fill in a questionnaire to assess demographic data and known STI risk factors. The factors assessed included the age of coitarche, the number of lifetime sexual partners, the number of sexual partners in the last year, a history of Pap smears, HPV vaccination and STIs. Methods of contraception, addictive substance usage, education level, marital status, original urban area and religion status were also evaluated.

HPV detection and genotyping were performed in the Laboratory of Molecular and Tumor Virology, Charles University, BIOCEV, Vestec, Czech Republic.

The tubes with samples and collecting tips were thoroughly vortexed to gain maximum cell load and pelleted by centrifugation. The dilution volume used was 200/400 µL depending on the size of the pellet. Isolation of the DNA was performed using the QIAamp DNA Mini Kit (Qiagen, Hilden, Germany) following the manufacturer’s instructions. DNA was eluted in 100 µL of AE buffer. The quality and quantity of DNA were measured spectrophotometrically (NanoDrop Technologies, Wilmington, DE). The volume was adjusted to achieve a maximum concentration of 50 ng/µL DNA.

The HPV detection was performed using commercial Anyplex II HPV 28 detection kit (Seegene, Seoul, South Korea). This multiplex real-time PCR-based method targets the L1 region of the viral genome and allows simultaneous genotyping of 28 HPV types and an internal control. Two PCR reactions were performed for each sample to detect the 19 HR HPV types (HPV 16, 18, 26, 31, 33, 35, 39, 45, 51, 52, 53, 56, 58, 59, 66, 68, 69, 73, 82) and the 9 LR HPV types (HPV 6, 11, 40, 42, 43, 44, 54, 61, 70). Negative and positive controls were included in all PCR runs.

Detection of *C. trachomatis* and *N. gonorrhoeae* was performed in the Department of Clinical Microbiology of the Military University Hospital Prague. Isolation of the DNA was carried out by an automated nucleic acid extracting device, Nextractor NX-48 system (Genolution, Seoul, South Korea), using 600 µL of lysis solution and 100 µL of elution solution per sample. Detection was performed by real-time PCR using GeneProof *C. trachomatis* and *N. gonorrhoeae* PCR Kit (Geneproof, Brno, Czech Republic).

### Statistical analysis

Statistical analysis was carried out using SPSS Statistics V.21. The Fisher exact test and standard χ^2^ test were used to determine the difference in the STI prevalence between the groups. A non-parametric approach was used to compare the difference in age between the groups due to abnormal age distribution and the small size of the groups. Marginal age values were not implemented, and direct adjustment for age using a study population was applied in order to exclude the differences in age structure between the groups. Bivariate analysis and Kruskal-Wallis test or χ^2^/Fisher exact test were used to evaluate if the groups differed in exposure to risk factors for STIs. For statistically significant results, the post hoc test or Bonferroni correction was applied. The level of definitive statistical significance was p<0.05.

## Results

A total of 141 women were enrolled in the pilot phase of the study: recruits (G1, n=72), active soldiers (G2, n=25) and control civilian group (G3, n=44). The median for age was 25.5 years in G1, 27 years in G2 and 28 years in G3. The difference in age median between groups G1 and G3 was statistically significant (p=0.007).

No *N. gonorrhoeae* case was found in the whole study population. The prevalence of *C. trachomatis* was 5.6% (4/72) in G1, 0% in G2 and 2.3% (1/44) in G3. There was no statistically significant difference between the groups (p=0.567).

**Figure 1:**
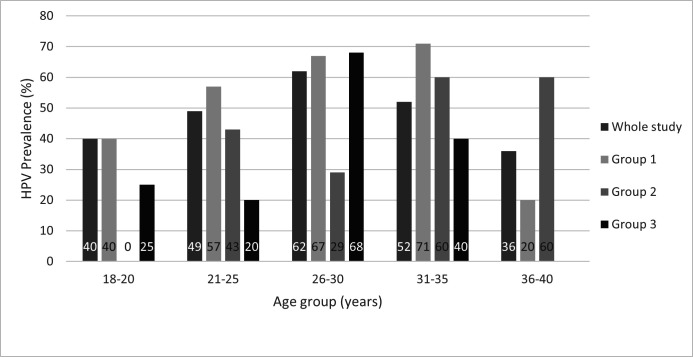
Prevalence of human papillomavirus DNA by age and study group.

The overall HPV prevalence in the studied participants was 51.8% (73/141), and it was age dependent across the study (figure 1).

The HPV prevalence in G1 was 55.6% (44/72), 48% (12/25) in G2 and 47.7% (21/44) in G3. After adjusting for age, the difference in HPV prevalence between G1 and both G2 and G3 was significant, 64.5 %, 46.4 %, and 47.3%, respectively (p=0.007 and p=0.01, respectively).

The prevalence of LR HPV types was 34.7% (25/72) in G1, 20% (5/25) in G2 and 22.7% (10/44) in G3. HR HPVs were more prevalent, 47.2% (34/72) in G1, 36% (9/25) in G2 and 36.4% (16/44) in G3. Differences between G1 compared with G2 and G3 in both LR HPV and HR HPV were statistically significant after adjustment for age. LR HPV prevalence in G1 was 39.8 %, 15.8% in G2 and 18.4% in G3 (p<0.001). HR HPV prevalence in G1 was 51.8 %, 29.8% in G2 and 28.6% in G3 (p<0.001; p=0.046, respectively). Infection with multiple HPV types (those in which two or more HPV types had been detected) was revealed in 43.0% (31/72) of G1, 20% (5/25) of G2 and 18.2% (8/44) of G3 participants. This shows a significantly higher prevalence of multiple HPV infection in G1 compared with G2 and G3 (p=0.040 and p=0.006, respectively). The most prevalent LR HPV type through the groups was HPV 42 (9.2 %). HPV 6 and 11 were each detected only once (0.7 %). In the case of HR HPV, the most frequent types were HPV 53 and 56 (both 9.2 %) with HPV 16, 58 and 59 (7.8 %) being less common (table 1).

**Table 1:** HPV genotype frequency detection

HPV genotype	All (n=141)		HPV vaccinated (n=53)		HPV non-vaccinated (n=43)	
	n	%	n	%	n	%
All HPV infections	73	51.8	29	54.7	43	49.4
HPV single infection	29	20.6	13	24.5	16	18.4
HPV multiple infections	44	31.2	16	30.2	28	32.2
HR HPV	59*	41.8	24	45.3	34	39.1
**16**	**11**	**7.8**	**1**	**1.9**	**10**	**11.5**
**18**	**5**	**3.6**	**1**	**1.9**	**4**	**4.6**
**31**	**7**	**5.0**	**2**	**3.8**	**5**	**5.8**
**33**	**2**	**1.4**	**0**	**0.0**	**2**	**2.3**
35	6	4.3	0	0.0	6	6.9
39	6	4.3	2	3.8	4	4.6
**45**	**2**	**1.4**	**1**	**1.9**	**1**	**1.2**
51	10*	7.1	5	9.4	4	4.6
**52**	**8**	**5.7**	**6**	**11.3**	**2**	**2.3**
53	13	9.2	6	11.3	7	8.1
56	13	9.2	6	11.3	7	8.1
**58**	11*	**7.8**	**4**	**7.6**	**6**	**6.9**
59	11*	7.8	3	5.7	7	8.1
66	8*	5.7	4	7.6	3	3.5
68	10*	7.1	5	9.4	4	4.6
73	2	1.4	0	0.0	2	2.3
82	4	2.8	2	3.8	2	2.3
LR HPV	40*	29.8	15	28.3	24	27.6
**6**	**1**	**0.7**	**1**	**1.9**	**0**	**0.0**
**11**	**1**	**0.7**	**0**	**0.0**	**1**	**1.2**
40	3	2.1	1	1.9	3	3.5
42	13*	9.2	7	13.2	5	5.8
43	5*	3.6	1	1.9	3	3.5
44	3*	2.1	1	1.9	1	1.2
54	9	6.4	2	3.8	7	8.1
61	2	1.4	1	1.9	1	1.2
70	4	2.8	3	5.7	1	1.2

Bold indicates HPV types preventable by vaccination.

* Patients with no answer in the questionnaire were not included in HPV vaccination statistics.

HR HPV, high-risk human papillomavirus; LR HPV, low-risk human papillomavirus.

Coitarche significantly differed for G1 with the lowest median at 16 years of age compared with the median of 17 years in G2 (p=0.005). The number of lifetime sexual partners and the number of sexual partners in the last year were not different between the groups (p=0.519 and p=0.114, respectively) (table 2).

**Table 2:** Analysis of ordinal STI risk factors in groups

Group		Age (years)	Coitarche (years)	Number of partners (n)	Partners in last year (n)
G1	Median	25.5	16.0	8.0	1.0
	Mean	25.8	16.4	9.7	1.8
	SD	5.9	1.6	8.9	1.7
	Range	18–40	14–22	1–50	0–10
	N	72	68	67	68
G2	Median	27.0	17.0	7.5	1.0
	Mean	28.5	17.7	9.3	1.5
	SD	5.8	2.1	6.8	1.2
	Range	20–39	15–25	2–30	0–6
	N	25	25	24	25
G3	Median	28	17	6	1
	Mean	27.8	17.4	9.3	1.4
	SD	6.1	2.2	9.0	1.1
	Range	19–35	14–23	1–40	0–6
	N	44	44	43	43
Kruskal-Wallis and post hoc tests	χ²	10.9	10.8	1.3	4.3
	df	2	2	2	2
	P value	0.004	0.005	0.519	0.114
	G1 and G2	0.081	0.005	X	X
	G1–G3	0.007	0.079	X	X
	G2 and G3	0.945	0.633	X	X

G1, recruits; G2, active soldiers; and G3, civilian control group.

df, degrees of freedom; N, number of subjects; STI, sexually transmitted infection.

Fewer women from G1 (47 %) reported living in a city larger than 100 thousand people compared with G2 (76 %) and G3 (69 %) members, and the difference was statistically significant (p=0.013) in contrast to the frequency of preventive Pap smear checks in the last 2 years (p=0.137), history of STIs (p=0.056), vaccination against HPV (p=0.751), usage of condom with a risky partner (p=0.573) or usage of contraception (p=0.664) where no statistically significant difference was found between the groups. Groups did not statistically differ in smoking (p=0.052) or alcohol consumption (p=0.393). The obtained data also did not show any difference in marital status (p=0.281), religious status (p=0.649) or education level (p=0.096) (table 3).

**Table 3:** Analysis of nominal STI risk factors in groups

^Risk factors^	^Category^	^G1n (%)^	^G2n (%)^	^G3n (%)^	^T value^	^df^	^P value^
^Regular Pap smear^	^0^	^9 (12.5)^	^5 (20.0)^	^2 (4.6)^	^3.979^	^2^	^0.137^
	^1^	^63 (87.5)^	^20 (80.0)^	^42 (95.5)^			
^History of STI^	^0^	^67 (94.4)^	^20 (80.0)^	^36 (81.8)^	^5.770^	^2^	^0.056^
	^1^	^4 (5.6)^	^5 (20.0)^	^8 (18.2)^			
^HPV vaccination^	^0^	^42 (59.2)^	^16 (64.0)^	^29 (65.9)^	^0.571^	^2^	^0.751^
	^1^	^29 (40.9)^	^9 (36.0)^	^15 (34.1)^			
^Condom usage in risky intercourse^	^0^	^6 (9.2)^	^3 (12.0)^	^7 (15.9)^	^1.113^	^2^	^0.573^
	^1^	^59 (90.8)^	^22 (88.0)^	^37 (84.1)^			
^Contraception^	^0^	^51 (71.8)^	^18 (72.0)^	^30 (68.2)^	^7.635^	^10^	^0.664^
	^Condom^	^1 (1.4)^	^2 (8.0)^	^1 (2.3)^			
	^Any other than condom^	^19 (26.8)^	^5 (20.0)^	^13 (29.6)^			
^Smoking^	^0^	^45 (67.2)^	^23 (92.0)^	^36 (85.7)^	^9.404^	^4^	^0.052^
	^1^	^22 (32.8)^	^2 (8.0)^	^6 (14.3)^			
^Addictive substances^	^0^	^66 (97.1)^	^25 (100.0)^	^42 (100.0)^	^2.000^	^2^	^0.368^
	^1^	^2 (2.9)^	^0 (0)^	^0 (0)^			
^Alcohol^	^0^	^64 (95.5)^	^25 (100.0)^	^39 (92.9)^	^1.869^	^2^	^0.393^
	^1^	^3 (4.5)^	^0 (0)^	^3 (7.1)^			
^Education level^	^Elementary school^	^4 (5.9)^	^0 (0)^	^1 (2.4)^	^10.775^	^6^	^0.096^
	^Vocational school^	^3 (4.4)^	^0 (0)^	^1 (2.4)^			
	^High school^	^35 (51.5)^	^12 (48.0)^	^12 (28.6)^			
	^University^	^26 (38.2)^	^13 (52.0)^	^28 (66.7)^			
^Basic medical education^	^0^	^61 (89.7)^	^15 (60.0)^	^37 (88.1)^	^12.687^	^2^	0.002
	^1^	^7 (10.29)^	^10 (40.0)^	^5 (11.9)^			
^Marital status^	^Single^	^57 (85.1)^	^21 (84.0)^	^34 (81.0)^	^5.059^	^4^	^0.281^
	^Married^	^8 (11.9)^	^2 (8.0)^	^8 (19.1)^			
	^Divorced^	^2 (3.0)^	^2 (8.0)^	^0 (0)^			
^City >100 thousand people^	^0^	^36 (52.9)^	^6 (24.0)^	^13 (31.0)^	^8.761^	^2^	0.013
	^1^	^32 (47.1)^	^19 (76.0)^	^29 (69.1)^			
^Religion (not specified)^	^0^	^63 (92.7)^	^22 (88.0)^	^37 (90.4)^	^4.205^	^6^	^0.649^
	^1^	^5 (7.4)^	^3 (12.0)^	^4 (9.8)^			

In the next step, the correlation between HPV and studied risk factors was analysed.

G1, recruits; G2, active soldiers; and G3, civilian control group. 0, negative answer (no); 1, positive answer (yes).

df, degrees of freedom; HPV, human papillomavirus; STI, sexually transmitted infection.

Promiscuity was confirmed to be a strong risk factor as women with three or more sexual partners in the last year were significantly more likely to test positive for HPV than those with two or less partners (59.5% (69/116) vs 5.6% (1/18); p=0.013). Early age of coitarche was also proved to be a risk factor. Lower age at the time of the first sexual intercourse was positively correlated with a higher prevalence of HPV infection (p=0.016). Marital status showed an association with the HPV prevalence. Married women had a lower chance of testing HPV positive than single or divorced women (22.2% (4/18) vs 56% (56/116), respectively; p=0.002). Concerning regular gynaecological examinations, women with a Pap smear in the last 2 years were more likely to test HPV positive (56% (70/125) compared with women without Pap smear 18.8% (3/16); p=0.006). For the rest of the risk factors mentioned above, no association with higher HPV prevalence has been proven. HPV vaccination as a protective factor was analysed only for HPV types included in all accessible vaccines (thus, types 6, 11, 16 and 18). Vaccinated women had lower HPV 16 prevalence compared with non-vaccinated (1.9% vs 11.5%) (p=0.040). There was no statistically significant difference in HPV 6, 11 and 18 prevalence between vaccinated and non-vaccinated women (p=0.379, p=1.000 and p=0.402, respectively) (table 2).

## Discussion

Answering the main question of the study, we did show a significant difference in the HPV prevalence between recruits (64.5 %) and both active soldiers (46.4 %) and civilian control group (47.3 %) after the direct age adjustment. Female recruits were also significantly more prone to multiple HPV-type infections. This is consistent with the work of Agan *et al* who showed that men entering army were in higher risk of testing seropositive for STIs.^12^

We did not find any difference in STI prevalence between active female soldiers and civilian women, and thus, we did not support the studies claiming a higher prevalence of STIs in soldiers.^9 12^ Even though the prevalence of *C. trachomatis* and HPV between the groups differed, low number of subjects seems to be the possible limitation for obtaining significant data.

The total HPV prevalence in the study was 51.8% with 47.7% for G3 alone. Taking into account a different age distribution, this is higher than the prevalence obtained in the Czech Republic screening population.^16^ There was a slight shift in age between the studies, considering the HPV peak prevalence. The studies did not differ in the HPV-type frequency with HPV 16 being the most frequent HR HPV and HPV 42 the most frequent LR HPV.

*C. trachomatis* prevalence in the general population of the Czech Republic is not known. There were 1803 reported cases in 2022, but no data on asymptomatic infections are available.^17^ The prevalence in this pilot study was 5.6% in G1, 0% in military G2 and 2.3% in G3 which stands for the civilian control group of women. Literature on the topic indicates a similar frequency of *C. trachomatis* in the general population in Central Europe. German study estimates the highest prevalence of 2.3% among 18–24-year-olds.^18^

Groups in our study differed in the age of coitarche. Women from G1 were more prone to start their sexual life at an earlier age. Taking this strong risk factor into consideration, this could possibly be the reason for the detected higher HPV prevalence in military recruits. Participants from G2 and G3 came from cities with over 100 thousand inhabitants more frequently compared with G1. Available data related to a possible association between age of coitarche and demographic urban characteristics are contradictory, and no clear link is confirmed.^19^,^21^

In our study, we have detected a significant association between an increased prevalence of HPV and a higher number (≥3) of sexual partners in the last year. An early start of the sexual life and the single or the single/divorced marital status had also increased the risk of HPV acquisition. All of these findings are consistent with the findings of other researchers.^22^,^24^ Women regularly undergoing Pap smear test were more likely to test HPV positive. This is rather surprising since we expect these women to have easier access to prevention and HPV vaccination. When researching HPV vaccination status among participants, we were not able to obtain detailed data on vaccine type and year of vaccination. Full statistics were performed only for the HPV types included in all the accessible HPV vaccines, thus HPV 6, 11, 16 and 18. Women vaccinated against HPV by any vaccine were less likely to be tested positive for HPV 16. No significant difference was proven for HPV 6, 11 and 18 between vaccinated and unvaccinated. This result could be explained by a lower frequency of the latter HPV types and limited study population.

This study had two main limitations. Regarding the sample size of the study, we managed to enrol 141 women across the studied groups with the smallest number of individuals (n=25) in the military. The second limitation is the significant age difference between the groups, leading to the necessity of direct age adjustment. The group size and unbalance in the age distribution should be targeted in the future phase of the study to enable more precise statistical analysis. A lower number of participants and refusal of the study can be caused by the necessity of a gynaecological examination to obtain cervical smears. New strategies including urine examination and self-collected vaginal samples should be considered to address the responders. Studies using the previously mentioned techniques performed mainly in the US military report higher numbers of participants.^911 12 25^,^27^ In comparison, European studies usually report data on smaller groups.^13 14^ In general, most of these studies refer to men or both genders together, missing the focus on women separately. Direct attention to female soldiers’ STI epidemiology is a strong point of this study.

## Conclusion

This prospective cross-sectional pilot study researched the prevalence of the most common STIs in the female Armed Forces personnel and the civilian population of the Czech Republic. No data on STI epidemiology in the Czech Republic military service are available since its foundation. We did observe higher HPV prevalence in military recruits compared with active female soldiers and civilian control group. No statistically significant difference was observed for *C. trachomatis* and *N. gonorrhoeae* between the military and civilian groups. Military recruits seem to be at higher risk of acquiring STI due to the more prevalent STI risk factors. This observation should be researched in an extended study to clarify the potential reproductive health risks under circumstances of subsequent military service. Preventive measures such as HPV vaccination or STI testing at the time of admission could be considered to actively reduce the risks of STI burden in the military.

## Data Availability

Data are available upon reasonable request.
